# Current Awareness on Comparative and Functional Genomics

**DOI:** 10.1002/cfg.59

**Published:** 2001-10


**Comparative and Functional Genomics**
*Comp. Funct. Genom.* 2001; **2:** 345–352
**DOI**:10.1002/cfg.59

### Current awareness on comparative and functional genomics

### 1 Reviews & symposia

Achard F, Vaysseix G, Barillot E*. 2001. *CRI Infobiogen, 523 Pl Terrasses Agora, FR-91000 Evry, France. XML, bioinformatics and data integration (Review). *Bioinformatics* **17:** (2) 115.

Aebersold R, Goodlett DR. 2001. Inst Syst Biol, 4225 Roosevelt Way NE, Seattle, Wa 98105, USA. Mass spectrometry in proteomics. *Chem Rev* **101:** (2) 269.

Al Lazikani B, Jung J, Xiang ZX, Honig B. 2001. Columbia Univ, Howard Hughes Med Inst, Dept Biochem & Mol Biophys, 630 West 168th St, New York, NY 10032, USA. Protein structure prediction. *Curr Opin Chem Biol* **5:** (1) 51.

Ali M, Markham AF, Isaacs JD. 2001. Univ Leeds, St James' Hosp, Mol Med Unit, Clin Sci Bldg, Leeds LS9 7TF, England. Application of differential display to immunological research. *J Immunol Methods* **250:** (1-2) 29.

Aravind L, Dixit VM, Koonin EV*. 2001. *NIH/Natl Lib Med, Natl Ctr Biotechnol Informat, Bethesda, Md 20894, USA. Apoptotic molecular machinery: Vastly increased complexity in vertebrates revealed by genome comparisons. *Science* **291:** (5507) 1279.

Ball CA, Cherry JM. 2001. Stanford Univ, Sch Med, Dept Genet, Stanford, Ca 94305, USA. Genome comparisons highlight similarity and diversity within the eukaryotic kingdoms. *Curr Opin Chem Biol* **5:** (1) 86.

Baner J, Nilsson M, Isaksson A, Mendel-Hartvig M, Antson DO, Landegren U*. 2001. *Rudbeck Lab, Dept Genet & Pathol, Beijer Lab, SE-75185 Uppsala, Sweden. More keys to padlock probes: Mechanisms for high-throughput nucleic acid analysis. *Curr Opin Biotechnol* **12:** (1) 11.

Barstead R. 2001. Oklahoma Med Res Fdn, 825 NE 13th St, Oklahoma City, Ok 73104, USA. Genome-wide RNAi. *Curr Opin Chem Biol* **5:** (1) 63.

Bashiardes S, Lovett M. 2001. Washington Univ, Sch Med, Dept Genet, Campus Box 8232, 4566 Scott Ave, St Louis, Mo 63110, USA. cDNA detection and analysis. *Curr Opin Chem Biol* **5:** (1) 15.

Becich MJ. 2000. UPMC Shadyside, Dept Pathol, 5230 Ctr Ave, Pittsburgh, Pa 15232, USA. The role of the pathologist as tissue refiner and data miner: The impact of functional genomics on the modern pathology laboratory and the critical roles of pathology informatics and bioinformatics. *Mol Diagn* **5:** (4) 287.

Berthet FX, Coche T*, Vinals C. 2001. *SmithKline Beecham Biol, 89 rue Inst, BE-1330 Rixensart, Belgium. Applied genome research in the field of human vaccines. *J Biotechnol* **85:** (2) 213.

Bevan M, Mayer K, White O, Eisen JA, Preuss D, Bureau T, Salzberg SL, Mewes HW. 2001. John Innes Ctr Plant Sci Res, Dept Mol Genet, Colney Lane, Norwich NR4 7UH, England. Sequence and analysis of the *Arabidopsis* genome. *Curr Opin Plant Biol* **4:** (2) 105.

Bichsel VE, Liotta LA, Petricoin EF*. 2001. *US/FDA, Ctr Biol Evaluat & Res, Div Therapeut Prod, Bethesda, Md 20892, USA. Cancer proteomics: From biomarker discovery to signal pathway profiling. *Cancer J* **7:** (1) 69.

Blohm DH, Guiseppi-Elie A. 2001. Univ Bremen, FB2-UFT, DE-28359 Bremen, Germany. New developments in microarray technology. *Curr Opin Biotechnol* **12:** (1) 41.

Bouche N, Bouchez D. 2001. Univ Leeds, Sch Biol, Ctr Plant Sci, Leeds LS2 9JT, England. *Arabidopsis* gene knockout: Phenotypes wanted. *Curr Opin Plant Biol* **4:** (2) 111.

Boulton SJ, Vincent S, Vidal M*. 2001. *Harvard Univ, Sch Med, Dana Farber Canc Inst, 44 Binney St, Boston, Ma 02115, USA. Use of protein-interaction maps to formulate biological questions. *Curr Opin Chem Biol* **5:** (1) 57.

Breyne P, Zabeau M*. 2001. *State Univ Ghent, Dept Plantengenet, Vlaams Interuniv Inst Biotechnol, Kl Ledeganckstr 35, BE-9000 Ghent, Belgium. Genome-wide expression analysis of plant cell cycle modulated genes. *Curr Opin Plant Biol* **4:** (2) 136.

Burke HB. 2000. New York Med Coll, Dept Med, Bioinformat & Computat Res Grp, Valhalla, NY 10595, USA. Discovering patterns in microarray data. *Mol Diagn* **5:** (4) 349.

Cahill DJ. 2001. Max-Planck-Inst Mol Genet, Ihnestr 73, DE-14195 Berlin, Germany. Protein and antibody arrays and their medical applications. *J Immunol Methods* **250:** (1-2) 81.

Carlson CS, Newman TL, Nickerson DA. 2001. Univ Washington, Dept Mol Biotechnol, Box 357330, Seattle, Wa 98195, USA. SNPing in the human genome. *Curr Opin Chem Biol* **5:** (1) 78.

Chich JF. 2001. INRA, Unite Rech Biochim & Struct Prot, Domaine de Vilvert, Bat 526, FR-78352 Jouy-en-Josas, France. A mini-review: Proteomic analysis, a post-genomic approach. *Lait* **81:** (1-2) 13.

Cho YR, Walbot V. 2001. Stanford Genome Technol Ctr, 855 Calif Ave, Palo Alto, Ca 94304, USA. Computational methods for gene annotation: The *Arabidopsis* genome. *Curr Opin Biotechnol* **12:** (2) 126.

Cobb JP, Brownstein NH, Watson MA, Shannon WD, Laramie JM, Qiu YY, Stormo GD, Morrissey JJ, Buchman TG, Karl IE, Hotchkiss RS. 2001. Washington Univ, Sch Med, Cellular Injury & Adaptat Lab, Injury Genomics Grp, Dept Surg, St Louis, Mo 63110, USA. Injury in the era of genomics (Review). *Shock* **15:** (3) 165.

Coulter LJ, Wright H, Reid HW*. 2001. *Moredun Res Inst, Int Res Ctr, Pentlands Sci Pk, Penicuik EH26 0PZ, Scotland. Molecular genomic characterization of the viruses of malignant catarrhal fever (Review). *J Comp Pathol* **124:** (1) 2.

Delneri D, Brancia FL, Oliver SG*. 2001. *Univ Manchester, Sch Biol Sci, 2.205 Stopford Bldg, Oxford Rd, Manchester M13 9PT, England. Towards a truly integrative biology through the functional genomics of yeast. *Curr Opin Biotechnol* **12:** (1) 87.

Diehn M, Relman DA. 2001. Stanford Univ, Dept Biochem, 269 Campus Dr, Stanford, Ca 94305, USA. Comparing functional genomic datasets: Lessons from DNA. microarray analyses of host-pathogen interactions. *Curr Opin Microbiol* **4:** (1) 95.

Dopazo J, Zanders E, Dragoni I, Amphlett C, Falciani F*. 2001. *Lorantis Ltd, Babraham Hall, Cambridge CB2 4UL, England. Methods and approaches in the analysis of gene expression data. *J Immunol Methods* **250:** (1-2) 93.

Dubey H, Grover A*. 2001. *Univ Delhi, Dept Plant Mol Biol, South Campus, Benito Juarez Rd, IN-110021 New Delhi, India. Current initiatives in proteomics research: The plant perspective. *Curr Sci India* **80:** (2) 262.

Dzau VJ, Mann MJ, Ehsan A, Griese DP. 2001. Harvard Univ, Brigham & Women's Hosp, Sch Med, Dept Med, Tower 1, 75 Francis St, Boston, Ma 02115, USA. Gene therapy and genomic strategies for cardiovascular surgery: The emerging field of surgiomics. *J Thorac Cardiovasc Surg* **121:** (2) 206.

Fagerlund TH, Braaten O. 2001. Univ Oslo, Inst Med Genet, POB 1036, NO-0315 Oslo, Norway. No pain relief from codeine ...? An introduction to pharmacogenomics (Review). *Acta Anaesthesiol Scand* **45:** (2) 140.

Fey SJ, Larsen PM. 2001. Univ Sthn Denmark, Ctr Proteome Anal, Int Sci Pk Odense, Forskerpk 108, DK-5230 Odense M, Denmark. 2D or not 2D. *Curr Opin Chem Biol* **5:** (1) 26.

Figeys D, Pinto D. 2001. MDS Ocata, 480 Univ Ave 4th Floor, Suite 401, Toronto, Ontario, Canada M5G 1V2. Proteomics on a chip:

In order to keep subscribers up-to-date with the latest developments in their field, this current awareness service is provided by John Wiley & Sons and contains newly-published material on comparative and functional genomics. Each bibliography is divided into 16 sections. 1 Reviews & symposia; 2 General; 3 Large-scale sequencing and mapping; 4 Evolutionary genomics; 5 Comparative genomics; 6 Pathways, gene families and regulons; 7 Pharmacogenomics; 8 Large-scale mutagenesis programmes; 9 Functional genomics; 10 Transcriptomics; 11 Proteomics; 12 Protein structural genomics; 13 Metabolomics; 14 Genomic approaches to development; 15 Technological advances; 16 Bioinformatics. Within each section, articles are listed in alphabetical order with respect to author. If, in the preceding period, no publications are located relevant to any one of these headings, that section will be omitted.

Copyright © 2001 John Wiley & Sons, Ltd.

Promising developments (Review). *Electrophoresis* **22:** (2) 208.
Fung ET, Thulasiraman V, Weinberger SR, Dalmasso EA. 2001. Ciphergen Biosyst, 6611 Dumbarton Circle, Fremont, Ca 94555, USA. Protein biochips for differential profiling. *Curr Opin Biotechnol* **12:** (1) 65.
Fussenegger M. 2001. ETH Zurich, Swiss Fed Inst Technol, Inst Biotechnol, CH-8093 Zurich, Switzerland. The impact of mammalian gene regulation concepts on functional genomic research, metabolic engineering, and advanced gene therapies (Review). *Biotechnol Prog* **17:** (1) 1.
Garesse R, Vallejo CG*. 2001. *Univ Autonoma Madrid, Fac Med, CSIC, Inst Invest Biomed Alberto Sols, Dept Bioquim, Arturo Duperier 4, ES-28029 Madrid, Spain. Animal mitochondrial biogenesis and function: A regulatory cross-talk between two genomes (Review). *Gene* **263:** (1-2) 1.
Graham MR, Smoot LM, Lei BF, Musser JM*. 2001. *NIH/NIAID, Rocky Mt Labs, Lab Human Bacterial Pathogenesis, 903 Sth 4th St, Hamilton, Mt 59840, USA. Toward a genome-scale understanding of group A *Streptococcus* pathogenesis. *Curr Opin Microbiol* **4:** (1) 65.
Grayhack EJ, Phizicky EM. 2001. Univ Rochester, Sch Med & Dent, Dept Biochem & Biophys, 601 Elmwood Ave, Box 712, Rochester, NY 14642, USA. Genomic analysis of biochemical function. *Curr Opin Chem Biol* **5:** (1) 34.
Green CD, Simons JF, Taillon BE, Lewin DA*. 2001. *Curagen Corp, Dept Gene Discovery, 555 Long Wharf Dr, New Haven, Ct 06511, USA. Open systems: Panoramic views of gene expression. *J Immunol Methods* **250:** (1-2) 67.
Hamer L, De Zwaan TM, Montenegro-Chamorro MV, Frank SA, Hamer JE*. 2001. *Paradigm Genet Inc, 104 Alexander Dr, Bldg 2, Res Triangle Pk, NC 27709, USA. Recent advances in large-scale transposon mutagenesis. *Curr Opin Chem Biol* **5:** (1) 67.
Herrmann JL, Rastelli L, Burgess CE, Fernandez EE, Rothberg BEG, Rothberg JM, Shimkets RA. 2001. Curagen Corp, Dept Internal Discovery, 555 Long Wharf Dr, New Haven, Ct 06511, USA. Implications of oncogenomics for cancer research and clinical oncology. *Cancer J* **7:** (1) 40.
Hirochika H. 2001. Natl Inst Agrobiol Resources, Dept Mol Genet, Tsukuba, Ibaraki 305 8602, Japan. Contribution of the Tos17 retrotransposon to rice functional genomics. *Curr Opin Plant Biol* **4:** (2) 118.
Hughes TR, Shoemaker DD. 2001. Rosetta Inpharmat, 12040 115th Ave NE, Kirkland, Wa 98034, USA. DNA microarrays for expression profiling. *Curr Opin Chem Biol* **5:** (1) 21.
Kuhn E. 2001. Univ Hohenheim 260, Inst Plant Physiol & Biotechnol, DE-70593 Stuttgart, Germany. From library screening to microarray technology: Strategies to determine gene expression profiles and to identify differentially regulated genes in plants (Review). *Ann Bot* **87:** (2) 139.
Kumar A, Snyder M*. 2001. *Yale Univ, Dept Mol Cellular & Dev Biol, POB 208103, New Haven, Ct 06520, USA. Emerging technologies in yeast genomics (Review). *Nat Rev Genet* **2:** (4) 302.
Kurland CG, Andersson SGE. 2000. Munkarpsv 21, SE-24332 Hoor, Sweden. Origin and evolution of the mitochondrial proteome. *Microbiol Mol Biol Rev* **64:** (4) 786.
Linton D, Karlyshev AV, Wren BW*. 2001. *Univ London, London Sch Hyg & Trop Med, Dept Infect & Trop Dis, Keppel St, London WC1E 7HT, England. Deciphering *Campylobacter jejuni* cell surface interactions from the genome sequence. *Curr Opin Microbiol* **4:** (1) 35.
Maheshwari SC, Maheshwari N, Sopory SK. 2001. Int Ctr Genet Engn & Biotechnol, POB 10504, Aruna Asaf Ali Rd, IN-110067 New Delhi, India. Genomics, DNA chips and a revolution in plant biology. *Curr Sci* **80:** (2) 252.
Maimone D, Dominici R, Grimaldi LME*. 2001. *Univ Milan, San Raffaele Sci Inst, Dept Neurol, Neuroimmunol Unit, DIBIT, via Olgettina 58, IT-20132 Milan, Italy. Pharmacogenomics of neurodegenerative diseases (Review). *Eur J Pharmacol* **413:** (1) 11.
Makarova KS, Aravind L, Wolf YI, Tatusov RL, Minton KW, Koonin EV, Daly MJ*. 2001. *Uniformed Serv Univ Hlth Sci, Dept Pathol, Room B3153, 4301 Jones Bridge Rd, Bethesda, Md 20814, USA. Genome of the extremely radiation-resistant bacterium *Deinococcus radiodurans* viewed from the perspective of comparative genomics. *Microbiol Mol Biol Rev* **65:** (1) 44.
Miller RA, Galecki A, Shmookler-Reis RJ. 2001. Univ Michigan, Dept Pathol, Box 0940, 1500 East Med Ctr Dr, Ann Arbor, Mi 48109, USA. Interpretation, design, and analysis of gene array expression experiments. *J Gerontol A Biol Sci Med* **56:** (2) B52.
Olden K, Guthrie J. 2001. NIH/NIEHS, Dept Hlth & Human Serv, POB 12233, Res Triangle Park, NC 27709, USA. Genomics: Implications for toxicology. *Mutat Res* **473:** (1) 3.
Pellegrini M. 2001. Protein Pathways, 1145 Gayley Ave, Suite 304, Los Angeles, Ca 90024, USA. Computational methods for protein function analysis. *Curr Opin Chem Biol* **5:** (1) 46.
Pickar D, Rubinow K. 2001. Comprehensive NeuroSci Inc, 4701 Willard Ave, Suite 105, Chevy Chase, Md 20815, USA. Pharmacogenomics of psychiatric disorders (Review). *Trends Pharmacol Sci* **22:** (2) 75.
Porubleva L, Chitnis PR*. 2000. *Iowa State Univ, Dept Biochem Biophys & Mol Biol, Ames, Ia 50011, USA. Proteomics: A powerful tool in the post-genomic era (Mini-Review). *Indian J Biochem Biophys* **37:** (6) 360.
Regnier F, Amini A, Chakraborty A, Geng M, Li JY, Riggs L, Sioma C, Wang ZSH, Zhang X. 2001. Purdue Univ, Dept Chem, West Lafayette, In 47904, USA. Multidimensional chromatography and the signature peptide approach to proteomics. *LC GC North Am* **19:** (2) 200.
Reineke U, Volkmer Engert R, Schneider Mergener J. 2001. Jerini AG, Rudower Chaussee 29, DE-12489 Berlin, Germany. Applications of peptide arrays prepared by the SPOT-technology. *Curr Opin Biotechnol* **12:** (1) 59.
Rimm DL, Camp RL, Charette LA, Costa J, Olsen DA, Reiss M. 2001. Yale Univ, Sch Med, Dept Pathol, 310 Cedar St, New Haven, Ct 06510, USA. Tissue microarray: A new technology for amplification of tissue resources. *Cancer J* **7:** (1) 24.
Rossignol M. 2001. INRA, UMR 5004, Pl Viala 2, FR-34060 Montpellier 1, France. Analysis of the plant proteome. *Curr Opin Biotechnol* **12:** (2) 131.
Sanseau P. 2001. GlaxoSmithKline, Target Bioinformatics, Gunnels Wood Rd, Stevenage SG1 2NY, England. Impact of human genome sequencing for *in silico* target discovery (Review). *Drug Discov Today,* **6:** (6) 316.
Sasaki T. 2001. Natl Inst Agrobiol Resources, Rice Genome Res Program, 1-2 Kannondai 2 chome, Tsukuba, Ibaraki 305 8602, Japan. The progress in rice genomics. *Euphytica* **118:** (2) 103.
Sigaux F. 2000. Hop St Louis, Inst Hematol, 1 Ave Claude Vellefaux, FR-75475 Paris, France. Cancer genomics or the molecular portraits of tumors. *Bull Acad Natl Med* **184:** (7) 1441.
Simone NL, Paweletz CP, Charboneau L, Petricoin EF, Liotta LA*. 2000. *NIH/NCI, Pathol Lab, Bldg 10, Room 2A33, 10 Ctr Dr, Bethesda, Md 20892, USA. Laser capture microdissection: Beyond functional genomics to proteomics. *Mol Diagn* **5:** (4) 301.
Stephens RS, Lammel CJ. 2001. Univ Calif Berkeley, Sch Publ Hlth, Div Infect Dis, Berkeley, Ca 94720, USA. Chlamydia outer membrane protein discovery using genomics. *Curr Opin Microbiol* **4:** (1) 16.
Tillib SV, Mirzabekov A*. 2001. *Russian Acad Sci, VA Engelhardt Mol Biol Inst, 32 Vavilov Str, RU-117984 Moscow, Russia. Advances in the analysis of DNA sequence variations using oligonucleotide microchip technology. *Curr Opin Biotechnol* **12:** (1) 53.
Tinsley C, Nassif X*. 2001. *Univ Paris 05, INSERM U411, Fac Med Necker Enfants Malad, FR-75730 Paris 15, France. Meningococcal pathogenesis: At the boundary between the pre- and post-genomic eras. *Curr Opin Microbiol* **4:** (1) 47.
Trethewey RN. 2001. Metanomics GmbH & Co KG, Tegeler Weg 33, DE-10589 Berlin, Germany. Gene discovery *via* metabolic profiling. *Curr Opin Biotechnol* **12:** (2) 135.
Triche TJ, Schofield D, Buckley J. 2001. Children's Hosp, Dept Pathol, 4650 Sunset Blvd, Los Angeles, Ca 90027, USA. DNA microarrays in pediatric cancer. *Cancer J* **7:** (1) 2.
Turner AJ, Isaac RE, Coates D. 2001. Univ Leeds, Sch Biochem & Mol Biol, Leeds LS2 9JT, England. The neprilysin (NEP) family of zinc metalloendopeptidases: Genomics and function (Review). *Bioessays* **23:** (3) 261.
Van Berkum NL, Holstege FCP. 2001. Univ Utrecht, Dept Med Genet, Med Ctr, Genomics Lab, POB 85060, NL-3508 AB Utrecht, The Netherlands. DNA microarrays: Raising the profile. *Curr Opin Biotechnol* **12:** (1) 48.
Vidan S, Snyder M*. 2001. *Yale Univ, Dept Mol Cellular & Dev Biol, POB 208103, New Haven, Ct 06520, USA. Large-scale mutagenesis: Yeast genetics in the genome era. *Curr Opin Biotechnol* **12:** (1) 28.
Westergren-Thorsson G, Malmstrom J, Marko-Varga G*. 2001. *AstraZeneca R&D Lund, Scheelevagen 8, SE-22187 Lund, Sweden. Proteomics: The protein expression technology to study connective tissue

Copyright © 2001 John Wiley & Sons, Ltd. 

biology. *J Pharmaceut Biomed Anal* **24:** (5-6) 815.

White BA, Morrison M. 2001. Univ Illinois, Dept Anim Sci, 1207 West Gregory Dr, Urbana, Il 61801, USA. Genomic and proteomic analysis of microbial function in the gastrointestinal tract of ruminants (Review). *Asian Australas J Anim Sci* **14:** (6) 880.

Yoshida M, Loo JA, Lepley RA*. 2001. *TIS Grp Inc, 200 Sth 6th St, Suite 450, Minneapolis, Mn 55402, USA. Proteomics as a tool in the pharmaceutical drug design process. *Curr Pharm Design* **7:** (4) 291.

Zhu H, Snyder M. 2001. Yale Univ, Dept Mol Cellular & Dev Biol, New Haven, Ct 06520, USA. Protein arrays and microarrays. *Curr Opin Chem Biol* **5:** (1) 40.

### 2 General

Ehrat M, Kresbach GM. 2001. Zeptosens AG, Benkenstr 254, CH-4018 Witterswil, Switzerland. DNA and protein microarrays and their contributions to proteomics and genomics. *Chimia* **55:** (1) 35.

### 3 Large-scale sequencing and mapping

Attoui H, Stirling JM, Munderloh UG, Billoir F, Brookes SM, Burroughs JN, De Micco P, Mertens PPC, De Lamballerie X*. 2001. *Fac Med Marseille, Unite Virus Emergents EA3292, Lab Virol Mol Trop & Transfus, 27 Blvd Jean Moulin, FR-13005 Marseille, France. Complete sequence characterization of the genome of the St Crois River virus, a new orbivirus isolated from cells of *Ixodes scapularis*. *J Gen Virol* **82:** (4) 795.

Cheung VG, Nowak N, Jang W, Kirsch IR, Zhao S, Chen XN, Furey TS, Kim UJ, Kuo WL, Olivier M *et al*. 2001. c/o Trask BJ, Fred Hutchinson Canc Res Ctr, 1100 Fairview Ave Nth, POB 19024, Seattle, Wa 98109, USA. Integration of cytogenetic landmarks into the draft sequence of the human genome. *Nature* **409:** (6822) 953.

Cole ST, Honore N, Eiglmeier K. 2000. Inst Pasteur, Unite Genet Mol Bacterienne, 28 rue Docteur Roux, FR-75724 Paris 15, France. Preliminary analysis of the genome sequence of *Mycobacterium leprae*. *Lepr Rev* **71:** (Suppl) S162.

Gockel G, Hachtel W*. 2000. *Univ Bonn, Inst Bot, Karlrobert Kreiten Str 13, DE-53115 Bonn, Germany. Complete gene map of the plastid genome of the nonphotosynthetic euglenoid flagellate *Astasia longa*. *Protist* **151:** (4) 347.

Gopal S, Schroeder M, Pieper U, Sczyrba A, Aytekin-Kurban G, Bekiranov S, Fajardo JE, Eswar N, Sanchez R, Sali A, Gaasterland T*. 2001. *Rockefeller Univ, Lab Computat Genomics, 1230 York Ave, New York, NY 10021, USA. Homology-based annotation yields 1,042 new candidate genes in the *Drosophila melanogaster* genome. *Nat Genet* **27:** (3) 337.

Guo H, Zhang J, Hu Y. 2000. Wuhan Univ, Inst Virol, CN-430072 Wuhan, Peoples Rep China. Complete sequence and organization of *Periplaneta fuliginosa* densovirus genome. *Acta Virol* **44:** (6) 315.

Kaneko T, Nakamura Y, Sato S, Asamizu E, Kato T, Sasamoto S, Watanabe A, Idesawa K, Ishikawa A, Kawashima K, Tabata S* *et al*. 2000. *Kazusa DNA Res Inst, 1532-3 Yana, Chiba 292 0812, Japan. Complete genome structure of the nitrogen-fixing symbiotic bacterium *Mesorhizobium loti*. *DNA Res* **7:** (6) 331.

Kaneko T, Nakamura Y, Sato S, Asamizu E, Kato T, Sasamoto S, Watanabe A, Idesawa K, Ishikawa A, Kawashima K, Tabata S* *et al*. 2000. *Address as above. Complete genome structure of the nitrogen-fixing symbiotic bacterium *Mesorhizobium loti* (supplement). *DNA Res* **7:** (6) 381.

Kato T, Kaneko T, Sato S, Nakamura Y, Tabata S*. 2000. *Address as above. Complete structure of the chloroplast genome of a legume, *Lotus japonicus*. *DNA Res* **7:** (6) 323.

Kelkar HS, Griffith J, Case ME, Covert SF, Hall RD, Keith CH, Oliver JS, Orbach MJ, Sachs MS, Wagner JR, Weise MJ, Wunderlich JK, Arnold J*. 2001. *Univ Georgia, Dept Genet, Athens, Ga 30602, USA. The *Neurospora crassa* genome: Cosmid libraries sorted by chromosome. *Genetics* **157:** (3) 979.

Lander ES, Linton LM, Birren B, Nusbaum C, Zody MC, Baldwin J, Devon K, Dewar K, Doyle M, FitzHugh W *et al*. 2001. Whitehead Inst Biomed Res, Ctr Genome Res, 9 Cambridge Ctr, Cambridge, Ma 02142, USA. Initial sequencing and analysis of the human genome. *Nature* **409:** (6822) 860.

Lilly JW, Havey MJ, Jackson SA, Jiang JM*. 2001. *Univ Wisconsin, Dept Hort, 1575 Linden Dr, Madison, Wi 53706, USA. Cytogenomic analyses reveal the structural plasticity of the chloroplast genome in higher plants. *Plant Cell* **13:** (2) 245.

May BJ, Zhang Q, Li LL, Paustian ML, Whittam TS, Kapur V*. 2001. *Univ Minnesota, Dept Vet Pathobiol, 1971 Commonwealth Ave, St Paul, Mn 55108, USA. Complete genomic sequence of *Pasteurella multocida*, Pm70. *Proc Natl Acad Sci U S A* **98:** (6) 3460.

McPherson JD, Marra M, Hillier L, Waterston RH, Chinwalla A, Wallis J, Sekhon M, Wylie K, Mardis ER, Wilson RK *et al*. 2001. Washington Univ, Sch Med, Genome Sequencing Ctr, Dept Genet, 4444 Forest Pk Blvd, St Louis, Mo 63108, USA. A physical map of the human genome. *Nature* **409:** (6822) 934.

Mo HD, Gu SL. 2001. Univ Yangzhou, Lab Quantitat Genet, CN-225009 Yangzhou, Peoples Rep China. Estimation of genome length. *Chin Sci Bull* **46:** (2) 122.

Nierman WC, Feldblyum TV, Laub MT, Paulsen IT, Nelson KE, Eisen J, Heidelberg JF, Alley MRK, Ohta N, Maddock JR *et al*. 2001. Inst Genomic Res, 9712 Med Ctr Dr, Rockville, Md 20850, USA. Complete genome sequence of *Caulobacter crescentus*. *Proc Natl Acad Sci U S A* **98:** (7) 4136.

Osoegawa K, Mammoser AG, Wu CY, Frengen E, Zeng CJ, Catanese JJ, De Jong PJ*. 2001. *Children's Hosp Oakland, Res Inst, 747 52nd St, Oakland, Ca 94609, USA. A bacterial artificial chromosome library for sequencing the complete human genome. *Genome Res* **11:** (3) 483.

Sachidanandam R, Weissman D, Schmidt SC, Kakol JM, Stein LD, Mullikin JC, Mortimore BJ, Willey DL, Hunt SE, Cole CG *et al*. 2001. c/o Altshuler D, 9 Cambridge Ctr, Cambridge, Ma 02139, USA. A map of human genome sequence variation containing 1.42 million single nucleotide polymorphisms. *Nature* **409:** (6822) 928.

Scheetz TE, Raymond MR, Nishimura DY, McClain A, Roberts C, Birkett C, Gardiner J, Zhang J, Butters N, Sun C, Kwitek-Black A, Jacob H, Casavant TL, Soares MB, Sheffield VC*. 2001. *Univ Iowa, Howard Hughes Med Inst, Iowa City, Ia 52242, USA. Generation of a high-density rat EST map. *Genome Res* **11:** (3) 497.

Shimizu T, Ohshima S, Ohtani K, Shimizu T, Hayashi H. 2001. Univ Tsukuba, Inst Basic Med Sci, Dept Microbiol, 1-1-1 Tenohdai, Tsukuba, Ibaraki 305 8575, Japan. Genomic map of *Clostridium perfringens* strain 13. *Microbiol Immunol* **45:** (2) 179.

Smith TPL, Grosse WM, Freking BA, Roberts AJ, Stone RT, Casas E, Wray JE, White J, Cho J, Fahrenkrug SC, Bennett GL, Heaton MP, Laegreid WW, Rohrer GA, Chitko-McKown CG, Pertea G, Holt I, Karamycheva S, Liang F, Quackenbush J, Keele JW. 2001. USDA/ARS, US Meat Anim Res Ctr, Clay Ctr, Ne 68933, USA. Sequence evaluation of four pooled-tissue normalized bovine cDNA libraries and construction of a gene index for cattle. *Genome Res* **11:** (4) 626.

Sugauchi F, Mizokami M*, Orito E, Ohno T, Kato H, Suzuki S, Kimura Y, Ueda R, Butterworth LA, Cooksley WGE. 2001. *Nagoya City Univ, Sch Med, Dept Lab Med II, Nagoya, Aichi 467 8601, Japan. A novel variant genotype C of hepatitis B virus identified in isolates from Australian Aborigines: Complete genome sequence and phylogenetic relatedness. *J Gen Virol* **82:** (4) 883.

Takano H, Abe T, Sakurai R, Moriyama Y, Miyazawa Y, Nozaki H, Kawano S, Sasaki N, Kuroiwa T. 2001. Kumamoto Univ, Fac Sci, Dept Biol Sci, Kumamoto 860 8555, Japan. The complete DNA sequence of the mitochondrial genome of *Physarum polycephalum*. *Mol Gen Genet* **264:** (5) 539.

Tu AHT, Voelker LL, Shen XJ, Dybvig K*. 2001. *Univ Alabama, Dept Comparat Med, Volker Hall, Room 418A, Birmingham, Al 35294, USA. Complete nucleotide sequence of the mycoplasma virus P1 genome. *Plasmid* **45:** (2) 122.

Venter JC, Adams MD, Myers EW, Li PW, Mural RJ, Sutton GG, Smith HO, Yandell M, Evans CA, Holt RA *et al*. 2001. Celera Genomics, 45 West Gude Dr, Rockville, 20850, USA. The sequence of the human genome. *Science* **291:** (5507) 1304.

Wiemann S, Weil B, Wellenreuther R, Gassenhuber J, Glassl S, Ansorge W, Bocher M, Blocker H, Bauersachs S, Blum H *et al*. 2001. German Canc Res Ctr, DE-69120 Heidelberg, Germany. Toward a catalog of human genes and proteins: Sequencing and analysis of 500 novel complete protein coding human cDNAs. *Genome Res* **11:** (3) 422.

Yu ZG, Anh VV, Wang B. 2001. Queensland Univ Technol, Ctr State Sci & Ind Math, GPO Box 2434, Brisbane, Qld 4001, Australia. Correlation property of length sequences based on global structure of the complete genome. *Phys Rev E* **6301:** (1) 1903.

Copyright © 2001 John Wiley & Sons, Ltd. 

### 4 Evolutionary genomics

Harrison PM, Echols N, Gerstein MB*. 2001. *Yale Univ, Dept Mol Biophys & Biochem, 260 Whitney Ave, POB 208114, New Haven, Ct 06511, USA. Digging for dead genes: An analysis of the characteristics of the pseudogene population in the *Caenorhabditis elegans* genome. *Nucleic Acids Res* **29:** (3) 818.

Jordan IK, Makarova KS, Spouge JL, Wolf YI, Koonin EV*. 2001. *NIH, Natl Ctr Biotechnol Informat, Natl Lib Med, Bethesda, Md 20894, USA. Lineage-specific gene expansions in bacterial and archeal genomes. *Genome Res* **11:** (4) 555.

Mazumder R, Kolaskar A, Seto D*. 2001. *George Mason Univ, Sch Computat Sci, 10900 Univ Blvd, Manassas, Va 20110, USA. GeneOrder: Comparing the order of genes in small genomes. *Bioinformatics* **17:** (2) 162.

Xie T, Ding DF*. 2000. *Chinese Acad Sci, Inst Biol Sci, Inst Biochem & Cell Biol, CN-200031 Shanghai, Peoples Rep China. Investigating 42 candidate orthologous protein groups by molecular evolutionary analysis on genome scale. *Gene* **261:** (2) 305.

### 5 Comparative genomics

Chopin A, Bolotin A, Sorokin A, Ehrlich SD, Chopin MC*. 2001. *CRJ INRA, Genet Microbienne, Domain Vilvert, FR-78352 Jouy-en-Josas, France. Analysis of six prophages in *Lactococcus lactis* IL1403: Different structure of temperate and virulent phage populations. *Nucleic Acids Res* **29:** (3) 644.

Dong YM, Glasner JD, Blattner FR, Triplett EW*. 2001. *Univ Wisconsin, Dept Agron, 1575 Linden Dr, Madison, Wi 53706, USA. Genomic interspecies microarray hybridization: Rapid discovery of three thousand genes in the maize endophyte, *Klebsiella pneumoniae* 342, by microarray hybridization with *Escherichia coli* K-12 open reading frames. *Appl Environ Microbiol* **67:** (4) 1911.

Gentles AJ, Karlin S*. 2001. *Stanford Univ, Dept Math, Stanford, Ca 94305, USA. Genome-scale compositional comparisons in eukaryotes. *Genome Res* **11:** (4) 540.

Kato-Maeda M, Rhee JT, Gingeras TR, Salamon H, Drenkow J, Smittipat N, Small PM*. 2001. *Stanford Univ, Med Ctr, 300 Pasteur Dr, Grant Bldg S-143, Stanford, Ca 94305, USA. Comparing genomes within the species *Mycobacterium tuberculosis*. *Genome Res* **11:** (4) 547.

Kirillova OV. 2001. St Petersburg State Univ, Dept Theoret Phys, Ulyanovskaya St 1, RU-198904 St Petersburg, Russia. Comparative statistical analysis of bacteria genomes in "word" context. *Physica A* **290:** (3-4) 453.

Lin LF, Posfai J, Roberts RJ, Kong HM*. 2001. *New England Biolabs Inc, 32 Tozer Rd, Beverly, Ma 01915, USA. Comparative genomics of the restriction-modification systems in *Helicobacter pylori*. *Proc Natl Acad Sci U S A* **98:** (5) 2740.

Ohshima K, Ando T, Motomura N, Matsuo K, Sako N. 2000. Saga Univ, Fac Agr, Lab Plant Virol, 1 Banchi, Honjo, Saga 840 8502, Japan. Comparative study on genomes of two Japanese melon necrotic spot virus isolates. *Acta Virol* **44:** (6) 309.

Porcella SF, Schwan TG*. 2001. *NIH/NIAID, Rocky Mt Labs, Lab Human Bacterial Pathogenesis, 903 Sth 4th St, Hamilton, Mt 59840, USA. *Borrelia burgdorferi* and *Treponema pallidum*: A comparison of functional genomics, environmental adaptations and pathogenic mechanisms. *J Clin Invest* **107:** (6) 651.

Quiros CF, Grellet F, Sadowski J, Suzuki T, Li G, Wroblewski T. 2001. Univ Calif, Dept Vegetable Crops, Davis, Ca 05616, USA. *Arabidopsis* and *Brassica* comparative genomics: Sequence, structure and gene content in the *ABI1-Rps2-Ck1* chromosomal segment and related regions. *Genetics* **157:** (3) 1321.

Tzung KW, Williams RM, Scherer S, Federspiel N, Jones T, Hansen N, Bivolarevic V, Huizar L, Komp C, Surzycki R, Tamse R, Davis RW, Agabian N*. 2001. *Univ Calif, Grad Program Oral Biol, San Francisco, Ca 94143, USA. Genomic evidence for a complete sexual cycle in *Candida albicans. Proc Natl Acad Sci U S A* **98:** (6) 3249.

Yuan SS, Mickelson D, Murtaugh MP, Faaberg KS*. 2001. *Univ Minnesota, Dept Vet Pathobiol, 205 Vet Sci Bldg, 1971 Commonwealth Ave, St Paul, Mn 55108, USA. Complete genome comparison of porcine reproductive and respiratory syndrome virus parental and attenuated strains. *Virus Res* **74:** (1-2) 99.

### 6 Pathways, gene families and regulons

Bornke F, Hajirezaei M, Sonnewald U. 2001. Inst Pflanzengenet & Kulturpflanzenforsch, Corrensstr 3, DE-06466 Gatersleben, Germany. Cloning and characterization of the gene cluster for palatinose metabolism from the phytopathogenic bacterium *Erwinia rhapontici*. *J Bacteriol* **183:** (8) 2425.

Coulson RMR, Enright AJ, Ouzounis CA. 2001. European Bioinformatics Inst, Res Programme, Computat Genomics Grp, EMBL Cambridge Outstn, Cambridge CB10 1SD, England. Transcription-associated protein families are primarily taxon-specific. *Bioinformatics* **17:** (1) 95.

Ermolaeva MD, White O, Salzberg SL. 2001. Inst Genomic Res, 9712 Med Ctr Dr, Rockville, Md 20850, USA. Prediction of operons in microbial genomes. *Nucleic Acids Res* **29:** (5) 1216.

Jiang SM, Wang L, Reeves PR*. 2000. *Univ Sydney, Dept Microbiol, Sydney, NSW 2006, Australia. Molecular characterization of *Streptococcus pneumoniae* type 4, 6B, 8, and 18C capsular polysaccharide gene clusters. *Infect Immun* **69:** (3) 1244.

McCue LA, Thompson W, Carmack CS, Ryan MP, Liu JS, Derbyshire V, Lawrence CE*. 2001. *New York State Dept Hlth, Wadsworth Ctr Labs & Res, Albany, NY 12201, USA. Phylogenetic footprinting of transcription factor building sites in proteobacterial genomes. *Nucleic Acids Res* **29:** (3) 774.

Nishida H. 2001. Univ Tokyo, Inst Mol & Cellular Biosci, Tokyo 113 0032, Japan. Distribution of genes for lysine biosynthesis through the aminoadipate pathway among prokaryotic genomes. *Bioinformatics* **17:** (2) 189.

Tan K, Moreno-Hagelsieb G, Collado-Vides J, Stormo GD*. 2001. *Washington Univ, Sch Med, Dept Genet, St Louis, Mo 63110, USA. A comparative genomics approach to prediction of new members of regulons. *Genome Res* **11:** (4) 566.

### 7 Pharmacogenomics

Achary MP, Jaggernauth W, Gross E, Alfieri A, Klinger HP, Vikram B. 2000. Yeshiva Albert Einstein Coll Med, Dept Radiat Oncol, 1300 Morris Pk Ave, Bronx, NY 10461, USA. Cell lines from the same cervical carcinoma but with different radiosensitivities exhibit different cDNA microarray patterns of gene expression. *Cytogenet Cell Genet* **91:** (1-4) 39.

Brutsche MH, Brutsche IC, Wood P, Brass A, Morrison N, Rattay M, Mogulkoc N, Simler N, Craven M, Custovic A, Egan JJG, Woodcock A. 2001. Univ Basel Hosp, Petersgraben 4, CH-4031 Basel, Switzerland. Apoptosis signals in atopy and asthma measured with cDNA arrays. *Clin Exp Immunol* **123:** (2) 181.

Cacabelos R, Alvarez A, Fernandez-Novoa L, Lombardi VRM. 2000. EuroEspes Biomed Res Ctr, Inst CNS Disorders, ES-15166 La Coruna, Spain. A pharmacogenomic approach to Alzheimer's disease. *Acta Neurol Scand* **102:** (Suppl 176) 12.

Chen CC, Shieh B, Jin YT, Liau YE, Huang CH, Liou JT, Wu LW, Huang WY, Young KC, Lai MD, Liu HS, Li C*. 2001. *Chung Shan Dent & Med Coll, Dept Med, Microbiol & Immunol Sect, 110, Sec 1 Chen Kuo Nth Rd, Taichung 402, Taiwan. Microarray profiling of gene expression patterns in bladder tumor cells treated with genistein. *J Biomed Sci* **8:** (2) 214.

Chen H, Liu J, Merrick BA, Waalkes MP*. 2001. *NIEHS, Comparat Carcinogenesis Lab, Mail Drop F0-09, 111 Alexander Dr, Res Triangle Pk, NC 27709, USA. Genetic events associated with arsenic-induced malignant transformation: Application of cDNA microarray technology. *Mol Carcinog* **30:** (2) 79.

Dales JP, Plumas J, Palmerini F, Devilard E, Defrance T, Lajmanovich A, Pradel V, Birg F, Xerri L*. 2001. *Inst J Paoli-Calmettes, Dept Pathol, IFR 57, 232 Blvd St Marguerite, BP 156, FR-13273 Marseille 9, France. Correlation between apoptosis macroarray gene expression profiling and histopathological lymph node lesions. *J Clin Pathol-Mol Pathol* **54:** (1) 17.

Hedenfalk I, Duggan D, Chen YD, Radmacher M, Bittner M, Simon R, Meltzer P, Gusterson B, Esteller M, Kallioniemi OPl, Trent J*. 2001. *NIH/NHGRI, Cancer Genet Branch, Bldg 49, Rm 4A22, Bethesda, Md 20892, USA. Gene-expression profiles in hereditary breast cancer. *N Engl J Med* **344:** (8) 539.

Kettunen E, Nissen AM, Ollikainen T, Taavitsainen M, Tapper J, Matt-

Copyright © 2001 John Wiley & Sons, Ltd. 

son K, Linnainmaa K, Knuutila S*, El Rifai W. 2001. *Univ Helsinki, Cent Hosp, Dept Med Genet, POB 404, Haartmanink 3, 4th Floor, FI-00029 Helsinki, Finland. Gene expression profiling of malignant mesothelioma cell lines: cDNA array study. *Int J Cancer* **91:** (4) 492.

Markert JM, Fuller CM, Gillespie GY, Bubien JK, McLean LA, Hong RL, Lee K, Gullans SR, Mapstone TB, Benos DJ*. 2001. *Univ Alabama, Dept Physiol & Biophys, 1918 Univ Blvd, Birmingham, Al 35294, USA. Differential gene expression profiling in human brain tumors. *Physiol Genomics* **5:** (1) 21.

Okabe H, Satoh S, Kato T, Kitahara O, Yanagawa R, Yamaoka Y, Tsunoda T, Furukawa Y, Nakamura Y*. 2001. *Univ Tokyo, Inst Med Sci, Ctr Human Genome, Mol Med Lab, Minato ku, 4-6-1 Shirokanedai, Tokyo 108 8639, Japan. Genome-wide analysis of gene expression in human hepatocellular carcinomas using cDNA microarray: Identification of genes involved in viral carcinogenesis and tumor progression. *Cancer Res* **61:** (5) 2129.

Okuno K, Yasutomi M, Nishimura N, Arakawa T, Shiomi M, Hida J, Ueda K, Minami K. 2001. Kinki Univ, Sch Med, Dept Surg I, 377-2 Ohno higashi, Osaka 589 8511, Japan. Gene expression analysis in colorectal cancer using practical DNA array filter. *Dis Colon Rectum* **44:** (2) 295.

Rappuoli R. 2001. Chiron SpA, IRIS, via Fiorentina 1, IT-53100 Siena, Italy. Reverse vaccinology, a genome-based approach to vaccine development. *Vaccine* **19:** (17-19) 2688.

Reilly TP, Bourdi M, Brady JN, Pise-Masision CA, Radonovich MF, George JW, Pohl LR. 2001. NIH/NHLBI, Mol & Cellular Toxicol Sect, Lab Mol Immunol, 9000 Rockville Pike, Bldg 10, Rm 8N110, Bethesda, Md 20892, USA. Expression profiling of acetaminophen liver toxicity in mice using microarray technology. *Biochem Biophys Res Commun* **282:** (1) 321.

Schuckit MA, Edenberg HJ, Kalmijn J, Flury L, Smith TL, Reich T, Bierut L, Goate A, Foroud T. 2001. Univ Calif, Dept Psychiat, 3350 La Jolla Village Dr, San Diego, Ca 92161, USA. A genome-wide search for genes that relate to a low level of response to alcohol. *Alcohol Clin Exp Res* **25:** (3) 323.

Vinals C, Gaulis S, Coche T. 2001. Glaxo SmithKline Biol, 89 rue Inst, BE-1330 Rixensart, Belgium. Using *in silico* transcriptomics to search for tumor-associated antigens for immunotherapy. *Vaccine* **19:** (17-19) 2607.

Wang E, Marincola FM*. 2001. *NIH/NCI, Surg Branch, Div Clin Sci, Ctr Clin, Bethesda, Md 20892, USA. cDNA arrays and the enigma of melanoma immune responsiveness. *Cancer J* **7:** (1) 16.

Westin L, Miller C*, Vollmer D, Canter D, Radtkey R, Nerenberg M, O'Connell JP. 2001. *Nanogen Inc, Assay Dev, 10398 Pacific Ctr Ct, San Diego, Ca 92121, USA. Antimicrobial resistance and bacterial identification utilizing a microelectronic chip array. *J Clin Microbiol* **39:** (3) 1097.

Wizemann TM, Heinrichs JH, Adamou JE, Erwin AL, Kunsch C, Choi GH, Barash SC, Rosen CA, Masure HR, Tuomanen E *et al*. 2000. c/o Koenig S, MedImmune Inc, 35 West Watkins Mill Rd, Gaithersburg, Md 20878, USA. Use of a whole genome approach to identify vaccine molecules affording protection against *Streptococcus pneumoniae* infection. *Infect Immun* **69:** (3) 1593.

Wong KK, Cheng RS, Mok SC. 2001. Pacific NW Natl Labs, Mol Biosci, 902 Battelle Blvd, Richland, Wa 99352, USA. Identification of differentially expressed genes from ovarian cancer cells by Micromax™ cDNA microarray system. *Biotechniques* **30:** (3) 670.

Zohlnhofer D, Klein CA, Richter T, Brandl R, Murr A, Nuhrenberg T, Schomig A, Baeuerle PA, Neumann FJ. 2001. Tech Univ Munich, Med Klin 1, Lazarettstr 36, DE-80636 Munich, Germany. Gene expression profiling of human stent-induced neointima by cDNA array analysis of microscopic specimens retrieved by helix cutter atherectomy: Detection of FK506-binding protein 12 upregulation. *Circulation* **103:** (10) 1396.

### 9 Functional genomics

Anholt RRH, Fanara JJ, Fedorowicz GM, Ganguly I, Kulkarni NH, Mackay TFC, Rollmann SM. 2001. Nth Carolina State Univ, WM Keck Ctr Behav Biol, Dept Zool, Box 7617, Raleigh, NC 27695, USA. Functional genomics of odor-guided behavior in *Drosophila melanogaster*. *Chem Senses* **26:** (2) 215.

Bachem C, Van der Hoeven R, Lucker J, Oomen R, Casarini E, Jacobsen E, Visser R. 2000. Univ Wageningen & Res Ctr, Dept Plant Sci, Lab Plant Breeding, POB 386, NL-6700 AJ Wageningen, The Netherlands. Functional genomic analysis of potato tuber life-cycle. *Potato Res* **43:** (4) 297.

De Backer MD, Nelissen B, Logghe M, Viaene J, Loonen I, Vandoninck S, De Hoogt R, Dewaele S, Simons FA, Verhasselt P, Vanhoof G, Contreras R, Luyten WH. 2001. Janssen Pharmaceut, Dept Adv Biotechnol, Turnhoutseweg 30, BE-2340 Beerse, Belgium. An antisense-based functional genomics approach for identification of genes critical for growth of *Candida albicans*. *Nat Biotechnol* **19:** (3) 235.

Florell SR, Coffin CM, Holden JA, Zimmermann JW, Gerwels JW, Summers BK, Jones DA, Leachman SA*. 2001. *Univ Utah, Hlth Sci Ctr, Dept Dermatol, 50 Nth Med Dr, Salt Lake City, Ut 84132, USA. Preservation of RNA for functional genomic studies: A multidisciplinary tumor bank protocol. *Mod Pathol* **14:** (2) 116.

Stevenson LF, Kennedy BK, Harlow E. 2001. MGH, Ctr Canc, Bldg 149, 13th St, Charlestown, Ma 02129, USA. A large-scale overexpression screen in *Saccharomyces cerevisiae* identifies previously uncharacterized cell cycle genes. *Proc Natl Acad Sci U S A* **98:** (7) 3946.

Wolf YI, Rogozin IB, Kondrashov AS, Koonin EV*. 2001. *NIH, Natl Ctr Biotechnol Informat, Natl Lib Med, Bethesda, Md 20894, USA. Genome alignment, evolution of prokaryotic genome organization, and prediction of gene function using genomic context. *Genome Res* **11:** (3) 356.

Yazaki J, Kishimoto N, Nakamura K, Fujii F, Shimbo K, Otsuka Y, Wu JZ, Yamamoto K, Sakata K, Sakaki T, Kikuchi S*. 2000. *Natl Inst Agrobiol Resources, 2-1-2 Kannondai, Tsukuba, Ibaraki 305 8602, Japan. Embarking on rice functional genomics via cDNA microarray: Use of 3′ UTR probes for specific gene expression analysis. *DNA Res* **7:** (6) 367.

### 10 Transcriptomics

Ang S, Lee CZ, Peck K, Sindici M, Matrubutham U, Gleeson MA, Wang JT*. 2000. *Natl Taiwan Univ, Coll Med, Dept Microbiol, Grad Inst Microbiol, 1 Sec 1 Jen Ai Rd, Taipei, Taiwan. Acid-induced gene expression in *Helicobacter pylori*: Study in genomic scale by microarray. *Infect Immun* **69:** (3) 1679.

Barrans JD, Stamatiou D, Liew CC. 2001. Harvard Univ, Brigham & Women's Hosp, Sch Med, Cardiovasc Genome Unit, Boston, Ma 02115, USA. Construction of a human cardiovascular cDNA microarray: Portrait of the failing heart. *Biochem Biophys Res Commun* **280:** (4) 964.

Brenner V, Lindauer K, Parkar A, Fordham J, Hayes I, Stow M, Gama R, Pollock K, Jupp R*. 2001. *Inpharmatica Ltd, 60 Charlotte St, London W1P 2AX, England. Analysis of cellular adhesion by microarray expression profiling. *J Immunol Methods* **250:** (1-2) 15.

Caron H, Van Schaik B, Ver der Mee M, Baas F, Riggins G, Van Sluis P, Hermus MC, Van Asperen R, Boon K, Voute PA, Heisterkamp S, Van Kampen A, Versteeg R. 2001. Emma Children's Hospital, Acad Med Ctr, Department Human Genet, POB 22700, NL-1100 DE Amsterdam, The Netherlands. The human transcriptome map: Clustering of highly expressed genes in chromosomal domains. *Science* **291:** (5507) 1289.

Chow ML, Moler EJ, Mian IS*. 2001. *Univ Calif, Lawrence Berkeley Lab, Div Cell & Mol Biol, Div Life Sci, Berkeley, Ca 94720, USA. Identifying marker genes in transcription profiling data using a mixture of feature relevance experts. *Physiol Genomics* **5:** (2) 99.

Coombes BK, Mahony JB. 2000. St Joseph Hosp, Reg Virol & Chlamydiol Lab, Father Sean O'Sullivan Res Ctr, 50 Charlton Ave East, Hamilton, Ontario, Canada L8N 4A6. cDNA array analysis of altered gene expression in human endothelial cells in response to *Chlamydia pneumoniae* infection. *Infect Immun* **69:** (3) 1420.

De Kievit TR, Gillis R, Marx S, Brown C, Iglewski BH*. 2001. *Univ Rochester, Med Ctr, Dept Microbiol & Immunol, 601 Elmwood Ave, Box 672, Rochester, NY 14642, USA. Quorum-sensing genes in *Pseudomonas aeruginosa* biofilms: Their role and expression patterns. *Appl Environ Microbiol* **67:** (4) 1865.

Foury F, Talibi D. 2001. Unite Biochim Physiol, Pl Croix Sud 2-20, BE-1348 Louvain, Belgium. Mitochondrial control of iron homeostasis: A genome wide analysis of gene expression in a yeast frataxin-deficient strain. *J Biol Chem* **276:** (11) 7762.

Fukasawa T, Fukuma M, Yano K, Sakurai H. 2001. 2-8-2 Maehara nishi, Chiba 274 0825, Japan. A genome-wide analysis of transcrip-

Copyright © 2001 John Wiley & Sons, Ltd. 

tional effect of Gal11 in *Saccharomyces cerevisiae*: An application of "mini-array hybridization technique". *DNA Res* **8:** (1) 23.

Lin CT, Sargan DR. 2001. Natl Taiwan Univ, Dept Vet Med, 142 Chou San Rd, Taipei 106, Taiwan. Generation and analysis of canine retinal ESTs: Isolation and expression of retina-specific gene transcripts. *Biochem Biophys Res Commun* **282:** (2) 394.

Myasnikova E, Samsonova A, Kozlov K, Samsonova M, Reinitz J*. 2001. *CUNY Mt Sinai Sch Med, Dept Mol Biol & Biochem, Box 1020, 1 Gustave Levy Pl, New York, NT 10029, USA. Registration of the expression patterns of *Drosophila* segmentation genes by two independent methods. *Bioinformatics* **17:** (1) 3.

Robert-Nicoud M, Flahaut M, Elalouf JM, Nicod M, Salinas M, Bens M, Doucet A, Wincker P, Artiguenave F, Horisberger JD, Vandewalle A, Rossier BC*, Firsov D. 2001. *University Lausanne, Institute Pharmacol & Toxicol, 27 rue Bugnon, CH-1005 Lausanne, Switzerland. Transcriptome of a mouse kidney cortical collecting duct cell line: Effects of aldosterone and vasopressin. *Proc Natl Acad Sci U S A* **98:** (5) 2712.

Sasaki Y, Asamizu E, Shibata D, Nakamura Y, Kaneko T, Awai K, Masuda T, Shimada H, Takamiya K, Tabata S, Ohta H. 2000. *Tokyo Inst Technol, Grad Sch Biosci & Biotechnol, Yokohama, Kanagawa 226 850, Japan. Genome-wide expression-monitoring of jasmonate-responsive genes in *Arabidopsis* using cDNA arrays. *Biochem Soc Trans* **28:** (6) 863.

Schaffer R, Landgraf J, Accerbi M, Simon V, Larson M, Wisman E*. 2001. *Michigan State Univ, Dept Energy, Plant Res Lab, East Lansing, Mi 48824, USA. Microarray analysis of diurnal and circadian-regulated genes in *Arabidopsis*. *Plant Cell* **13:** (1) 113.

Seki M, Narusaka M, Abe H, Kasuga M, Yamaguchi-Shinozaki K, Carninci P, Hayashizaki Y, Shinozaki K*. 2001. *RIKEN, Genomic Sci Ctr, Plant Funct Genomics Res Group, Plant Mutation Exploration Team, 3-1-1 Koyadai, Tskuba, Ibaraki 305 0074, Japan. Monitoring the expression patterns of 1300 *Arabidopsis* genes under drought and cold stresses by using a full-length cDNA microarray. *Plant Cell* **13:** (1) 61.

Shoemaker DD, Schadt EE, Armour CD, He YD, Garrett-Engele P, McDonagh PD, Loerch PM, Leonardson A, Lum PY, Cavet G *et al.* 2001. c/o Boguski MS, Rosetta Inpharmatics Inc, 12040 115th Ave NE, Kirkland, Wa 98034, USA. Experimental annotation of the human genome using microarray technology. *Nature* **409:** (6822) 922.

Tsou R, Cole JK, Nathens AB, Isik FF, Heimbach DM, Engrav LH, Gibran NS*. 2000. *Univ Washington, Harborview Med Ctr, Burn Ctr, 325 9th Ave, Box 359796, Seattle, Wa 98104, USA. Analysis of hypertrophic and normal scar gene expression with cDNA microarrays. *J Burn Care Rehabil* **21:** (6) 541.

Wendisch VF, Zimmer DP, Khodursky A, Peter B, Cozzarelli N, Kustu S*. 2001. *Univ Calif, Dept Plant & Microbial Biol, 111 Koshland Hall, Berkeley, Ca 94720, USA. Isolation of *Escherichia coli* mRNA and comparison of expression using mRNA and total RNA on DNA microarrays. *Anal Biochem* **290:** (2) 205.

Yano N, Endoh M, Fadden KJ, Yamashita H, Sakai H, Kurokawa K, Abboud HE, Rifai A*. 2000. *Brown Univ, Rhode Island Hosp, Dept Pathol, 593 Eddy St, Providence, RI 02903, USA. Genomic repertoire of human mesangial cells: Comprehensive analysis of gene expression by cDNA array hybridization. *Nephrology* **5:** (4) 215.

Yoshida K, Kobayashi K, Miwa Y, Kang CM, Matsunaga M, Yamaguchi H, Tojo S, Yamamoto M, Nishi R, Ogasawara N, Nakayama T, Fujita Y*. 2001. *Fukuyama Univ, Fac Engn, Dept Biotechnol, 985 Sanzo, Higashimura cho, Hiroshima 729 0292, Japan. Combined transcriptome and proteome analysis as a powerful approach to study genes under glucose repression in *Bacillus subtilis*. *Nucleic Acids Res* **29:** (3) 683.

Zhu H, Nowrousian M, Kupfer D, Colot HV, Berrocal-Tito G, Lai HS, Bell-Pedersen D, Roe BA, Loros JJ, Dunlap JC*. 2001. *Dartmouth Coll Sch Med, Dept Genet, Hanover, NH 03755, USA. Analysis of expressed sequence tags from two starvation, time-of-day-specific libraries of *Neurospora crassa* reveals novel clock-controlled genes. *Genetics* **157:** (3) 1057.

### 11 Proteomics

Blee KA, Wheatley ER, Bonham VA, Mitchell GP, Robertson D, Slabas AR, Burrell MM, Wojtaszek P, Bolwell GP*. 2001. *Polish Acad Sci, Inst Bioorgan Chem, Noskowskiego 12-14, PL-61704 Poznan, Poland. Proteomic analysis reveals a novel set of cell wall proteins in a transformed tobacco cell culture that synthesizes secondary walls as determined by biochemical and morphological parameters. *Planta* **212:** (3) 404.

Brancia FL, Butt A, Beynon RJ, Hubbard SJ, Gaskell SJ, Oliver SG*. 2001. *Univ Manchester, Sch Biol Sci, 2.205 Stopford Bldg, Oxford Rd, Manchester M13 9PT, England. A combination of chemical derivatisation and improved bioinformatic tools optimises protein identification for proteomics. *Electrophoresis* **22:** (3) 552.

Hoang VM, Foulk R, Clauser K, Burlingame A, Gibson BW, Fisher SJ*. 2001. *Univ Calif, Dept Pharmaceut Chem, Grad Program Pharmaceut Chem, San Francisco, Ca 94143, USA. Functional proteomics: Examining the effects of hypoxia on the cytotrophoblast protein repertoire. *Biochemistry* **40:** (13) 4077.

Hu S, Lee R, Zhang Z, Krylov SN, Dovichi NJ*. 2001. *University Alberta, Department Chem, E3-44 Chem Bldg, Edmonton, Alberta, Canada T6G 2G2. Protein analysis of an individual *Caenorhabditis elegans* single-cell embryo by capillary electrophoresis. *J Chromatogr B* **752:** (2) 307.

Husi H, Ward MA, Choudhary JS, Blackstock WP, Grant SGN*. 2000. *Univ Edinburgh, Ctr Neurosci, Ctr Genome Res, West Mains Rd, Edinburgh EH9 3JQ, Scotland. Proteomic analysis of NMDA receptor-adhesion protein signalling complexes. *Nat Neurosci* **3:** (7) 661.

Kidd D, Liu YS, Cravatt BF*. 2001. *Scripps Clin & Res Inst, Skaggs Inst Chem Biol, 10550 Nth Torrey Pines Rd, La Jolla, Ca 92037, USA. Profiling serine hydrolase activities in complex proteomes. *Biochemistry* **40:** (13) 4005.

Marcotte EM. 2001. Univ Texas, Dept Chem & Biochem, Austin, Tx 78712, USA. Measuring the dynamics of the proteome. *Genome Res* **11:** (2) 191.

Morris AC, Djordjevic MA*. 2001. *Australian Natl Univ, Res Sch Biol Sci, Genomic Interact Grp, GPO Box 475, Canberra, ACT 2601, Australia. Proteome analysis of cultivar-specific interactions between *Rhizobium leguminosarum* biovar *trifolii* and subterranean clover cultivar Woogenellup. *Electrophoresis* **22:** (3) 586.

Pevzner PA, Mulyukov Z, Dancik V, Tang CL. 2001. Univ Calif San Diego, Dept Comp Sci & Engn, La Jolla, Ca 92093, USA. Efficiency of database search for identification of mutated and modified proteins *via* mass spectrometry. *Genome Res* **11:** (2) 290.

Ping PP, Zhang J, Pierce WM, Bolli R. 2001. Cardiol Res, Baxter Bldg, Suite 122, 570 Sth Preston St, Louisville, Ky 40202, USA. Functional proteomic analysis of protein kinase Cε signaling complexes in the normal heart and during cardioprotection. *Circ Res* **88:** (1) 59.

Sironi L, Tremoli E, Miller I, Guerrini U, Calvio AM, Eberini I, Gemeiner M, Asdente M, Paoletti R, Gianazza E*. 2001. *Univ Milan, Fac Farm, Dipt Sci Farmacol, via Balzaretti 9, IT-20133 Milan, Italy. Acute-phase proteins before cerebral ischemia in stroke-prone rats: Identification by proteomics. *Stroke* **32:** (3) 753.

Vido K, Spector D, Lagniel G, Lopez S, Toledano MB, Labarre J*. 2001. *CEA Saclay, Serv Biochim & Genet Mol, Bat 142, FR-91191 Gif-sur-Yvette, France. A proteome analysis of the cadmium response in *Saccharomyces cerevisiae*. *J Biol Chem* **276:** (11) 8469.

Washburn MP, Wolters D, Yates JR*. 2001. *Syngenta Agr Discovery Inst, 3115 Merryfield Row, Suite 100, San Diego, Ca 92121, USA. Large-scale analysis of the yeast proteome by multidimensional protein identification technology. *Nat Biotechnol* **19:** (3) 242.

### 12 Protein structural genomics

Brocchieri L. 2001. Stanford Univ, Dept Math, Stanford, Ca 94305, USA. Low-complexity regions in *Plasmodium* proteins: In search of a function. *Genome Res* **11:** (2) 195.

McGuffin LJ, Bryson K, Jones DT*. 2001. *Brunel Univ, Dept Biol Sci, Bioinformatics Grp, Uxbridge UB8 3PH, England. What are the baselines for protein fold recognition? *Bioinformatics* **17:** (1) 63.

Siddiqui AS, Dengler U, Barton GJ*. 2001. *EMBL, Eur Bioinformat Inst, Wellcome Trust Genome Campus, Cambridge CB10 1SD, England. 3Dee: A database of protein structural domains. *Bioinformatics* **17:** (2) 200.

Simons KT, Strauss C, Baker D*. 2001. *Univ Washington, Box 357350, Seattle, Wa 98195, USA. Prospects of *ab initio* protein structural genomics. *J Mol Biol* **306:** (5) 1191.

Stothard PM. 2001. Univ Alberta, Dept Biol Sci, Edmonton, Alberta,

Copyright © 2001 John Wiley & Sons, Ltd. 

Canada T6G 2E9. COMBOSA3D: combining sequence alignments with three-dimensional structures. *Bioinformatics* **17:** (2) 198.

### 13 Metabolomics

Roessner U, Luedemann A, Brust D, Fiehn O, Linke T, Willmitzer L, Fernie AR*. 2001. *Max-Planck-Inst Mol Pflanzenphysiol, Muhlenberg 1, DE-14476 Golm, Germany. Metabolic profiling allows comprehensive phenotyping of genetically or environmentally modified plant systems. *Plant Cell* **13:** (1) 11.

### 14 Genomic approaches to development

Beck GR, Zerler B, Moran E*. 2001. *Temple Univ, Sch Med, Fels Inst Canc Res & Mol Biol, Philadelphia, Pa 19140, USA. Gene array analysis of osteoblast differentiation. *Cell Growth Differ* **12:** (2) 61.

Benfey PN, Weigel D*. 2001. *Salk Institute Biol Studies, Plant Biol Lab, 10010 Nth Torrey Pines Rd, La Jolla, Ca 92037, USA. Transcriptional networks controlling plant development. *Plant Physiol* **125:** (1) 109.

Chiang LW, Grenier JM, Ettwiller L, Jenkins LP, Ficenec D, Martin J, Jin FY, Di Stefano PS, Wood A. 2001. Millennium Pharmaceut, 640 Mem Dr, Cambridge, Ma 02139, USA. An orchestrated gene expression component of neuronal programmed cell death revealed by cDNA array analysis. *Proc Natl Acad Sci U S A* **98:** (5) 2814.

Kushalappa KM, Mattoo AK, Vijayraghavan U*. 2000. *Indian Inst Sci, Dept Microbiol & Cell Biol, IN-560012 Bangalore, India. A spectrum of genes expressed during early stages of rice panicle and flower development. *J Genet* **79:** (2) 25.

Lawson A, Colas JF, Schoenwolf GC*. 2001. *Univ Utah, Sch Med, Dept Neurobiol & Anat, 50 Nth Med Dr, Salt Lake City, Ut 84132, USA. Classification scheme for genes expressed during formation and progression of the avian primitive streak. *Anat Rec* **262:** (2) 221.

Li XM, Mohan S, Gu WK, Baylink DJ*. 2001. *JL Pettis VA Med Ctr, Musculoskeletal Dis Ctr, Div Mol Genet, 11201 Benton St, Loma Linda, Ca 92357, USA. Analysis of gene expression in the wound repair/regeneration process. *Mamm Genome* **12:** (1) 52.

Welle S, Brooks A, Thornton CA. 2001. Univ Rochester, Med Ctr, Box 693, 601 Elmwood Ave, Rochester, NY 14642, USA. Senescence-related changes in gene expression in muscle: Similarities and differences between mice and men. *Physiol Genomics* **5:** (2) 67.

Wines ME, Lee L, Katari MS, Zhang LQ, De Rossi C, Shi Y, Perkins S, Feldman M, McCombie WR, Holdener BC*. 2001. *SUNY Stony Brook, Dept Biochem & Cell Biol, Inst Cell & Dev Biol, Stony Brook, NY 11794, USA. Identification of mesoderm development (*mesd*) candidate genes by comparative mapping and genome sequence analysis. *Genomics* **72:** (1) 88.

### 15 Technological advances

Bousse L, Mouradian S, Minalla A, Yee H, Williams K, Dubrow R. 2001. Caliper Technol Corporation, 605 Fairchild Drive, Mount View, Ca 94043, USA. Protein sizing on a microchip. *Anal Chem* **73:** (6) 1207.

Bratt C, Lindberg C, Marko-Varga G*. 2001. *AstraZeneca R&D, Scheelevagen 8, SE-22187 Lund, Sweden. Restricted access chromatographic sample preparation of low mass proteins expressed in human fibroblast cells for proteomics analysis. *J Chromatogr A* **909:** (2) 279.

Call DR, Chandler DP, Brockman F. 2001. Washington State Univ, Dept Vet Microbiol & Pathol, POB 647040, Pullman, Wa 99164, USA. Fabrication of DNA microarrays using unmodified oligonucleotide probes. *Biotechniques* **30:** (2) 368.

Chang HT, Yergey AL, Chrambach A*. 2001. *NIH/NICHHD, Lab Cellular & Mol Biophys, Macromol Anal Sect, Bldg 10, Rm 9D50, Bethesda, Md 20892, USA. Electroelution of proteins from bands in gel electrophoresis without gel sectioning for the purpose of protein transfer into mass spectrometry: Elements of a new procedure. *Electrophoresis* **22:** (3) 394.

Chong BE, Hamler RL, Lubman DM*, Ethier SP, Rosenspire AJ, Miller FR. 2001. *Univ Michigan, Dept Chem, Ann Arbor, Mi 48109, USA. Differential screening and mass mapping of proteins from premalignant and cancer cell lines using nonporous reversed-phase HPLC coupled with mass spectrometric analysis. *Anal Chem* **73:** (6) 1219.

Cox JM. 2001. Glaxo Wellcome Res & Dev Ltd, Med Res Ctr, Gunnels Wood Rd, Stevenage SG1 2NY, England. Applications of nylon membrane arrays to gene expression analysis. *J Immunol Methods* **250:** (1-2) 3.

Davis MT, Beierle J, Bures ET, McGinley MD, Mort J, Robinson JH, Spahr CS, Yu W, Luethy R, Patterson SD. 2001. Amgen Inc, Dept Biochem, Thousand Oaks, Ca 91320, USA. Automated LC-LC-MS-MS platform using binary ion-exchange and gradient reversed-phase chromatography for improved proteomic analyses. *J Chromatogr B* **752:** (2) 281.

Gao H, Oberringer M, Englisch A, Hanselmann RG, Hartmann U. 2001. Univ Saarland, Inst Expt Phys, POB 151150, DE-66041 Saarbrucken, Germany. The scanning near-field optical microscope as a tool for proteomics. *Ultramicroscopy* **86:** (1-2) 145.

Geng M, Zhang X, Bina M, Regnier F*. 2001. *Purdue Univ, Dept Chem, Brown Bldg, West Lafayette, In 47907, USA. Proteomics of glycoproteins based on affinity selection of glycopeptides from tryptic digests. *J Chromatogr B* **752:** (2) 293.

Gobom J, Schuerenberg M, Mueller M, Theiss D, Lehrach H, Nordhoff E. 2001. Max-Planck-Inst Mol Genet, Ihnestr 73, Berlin, Germany. α-Cyano-4-hydroxycinnamic acid affinity sample preparation: A protocol for MALDI-MS peptide analysis in proteomics. *Anal Chem* **73:** (3) 434.

Griffin TJ, Gygi SP, Rist B, Aebersold R, Loboda A, Jilkine A, Ens W, Standing KG. 2001. Inst Syst Biol, 4225 Roosevelt Way NW, Suite 200, Seattle, Wa 98105, USA. Quantitative proteomic analysis using a MALDI quadrupole time-of-flight mass spectrometer. *Anal Chem* **73:** (5) 978.

Hasan MT, Schonig K, Berger S, Graewe W, Bujard H*. 2001. *Univ Heidelberg, Zentrum Mol Biol, Neuenheimer Feld 282, DE-69120 Heidelberg, Germany. Long-term, noninvasive imaging of regulated gene expression in living mice. *Genesis* **29:** (3) 116.

Jankowski J, Stephan N, Knobloch M, Fischer S, Schmaltz D, Zidek W, Schluter H. 2001. Free Univ Berlin, Klin Benjamin Franklin, Med Klin IV, Hindenburgdamm 30, DE-12200 Berlin, Germany. Mass spectrometry-linked screening of protein fractions for enzymatic activities: A tool for functional genomics. *Anal Biochem* **290:** (2) 324.

Lamer S, Jungblut PR*. 2001. *Max-Planck-Inst Infect Biol, Cent Support Unit Biochem, Berlin, Germany. Matrix-assisted laser desorption/ionization mass spectrometry peptide mass fingerprinting for proteome analysis: Identification efficiency after on-blot or in-gel digestion with and without desalting procedures. *J Chromatogr B* **752:** (2) 311.

Naimuddin M, Kurazono T, Zhang YH, Watanabe T, Yamaguchi M, Nishigaki K*. 2000. *Saitama University, Dept Funct Mat Sci, 255 Shimo okubo, Urawa, Saitama 338 8570, Japan. Species-identification dots: A potent tool for developing genome microbiology. *Gene* **261:** (2) 243.

Poirier F, Imam N, Pontet M, Joubert-Caron R, Caron M*. 2001. *Univ Paris 13, UFR SMBH Leonard de Vinci, EA 1625, 74 rue Marcel Cachin, FR-93017 Bobigny, France. The BPP (protein biochemistry and proteomics) two-dimensional electrophoresis database. *J Chromatogr B* **753:** (1) 23.

Powell M, Tempst P*. 2001. *Mem Sloan Kettering Canc Ctr, Prot Ctr, 1275 York Ave, New York, NY 10021, USA. Microflow-based automated chemistries: Application to protein sequencing. *Anal Chem* **73:** (4) 776.

Rajeevan MS, Vernon SD, Taysavang N, Unger ER. 2001. US Dept HHS, Ctr Dis Control & Prevent, Natl Ctr Infect Dis, Div Viral & Rickettsial Dis, Atlanta, Ga 30332, USA. Validation of array-based gene expression profiles by real-time (kinetic) RT-PCR. *J Mol Diagn* **3:** (1) 26.

Shmelkov SV, Visser JWM, Belyavsky AV*. 2001. *New York Blood Ctr, 310 East 67th St, New York, NY 10021, USA. Two-dimensional gene expression fingerprinting. *Anal Biochem* **290:** (1) 26.

Smith RD. 2000. Pacific NW Natl Lab, Environm Mol Sci Lab, Mail Stop K8-98, POB 999, Richland, Wa 99352, USA. Evolution of ESI-mass spectrometry and Fourier transform ion cyclotron resonance for proteomics and other biological applications. *Int J Mass Spectrom* **200:** (1-3) 509.

Steen H, Mann M*. 2001. *Odense Univ, Univ Sthn Denmark, Dept Biochem & Mol Biol, Prot Interact Lab, Campusvej 55, DK-5230 Odense M, Denmark. Similarity between condensed phase and gas phase chemistry: Fragmentation of peptides containing oxidized cys-

Copyright © 2001 John Wiley & Sons, Ltd.

teine residues and its implications for proteomics. *J Am Soc Mass Spectrom* **12:** (2) 228.

Van de Rijke F, Zijlmans H, Li S, Vail T, Raap AK, Niedbala RS, Tanke HJ*. 2001. *Leiden Univ, Med Ctr, Dept Mol Cell Biol, Lab Cytochem & Cytomet, Leiden, The Netherlands. Up-converting phosphor reporters for nucleic acid microarrays. *Nat Biotechnol* **19:** (3) 273.

Werner T. 2001. Genomatix Software GmbH, Karlstr 55, DE-80333 Munich, Germany. Target gene identification from expression array data by promoter analysis. *Biomol Eng* **17:** (3) 87.

Yamamoto M, Wakatsuki T, Hada A, Ryo A. 2001. Natl Def Med Coll, Dept Biochem, 3-2 Namiki, Tokorozawa, Saitama 359 8513, Japan. Use of serial analysis of gene expression (SAGE) technology. *J Immunol Methods* **250:** (1-2) 45.

Youil R, Toner TJ, Su Q, Kaslow DC. 2001. Merck & Co Inc, Dept Virus & Cell Biol, West Point, Pa 19486, USA. Rapid method for the isolation of full length adenoviral genomes by bacterial intermolecular homologous recombination. *J Virol Methods* **92:** (1) 91.

Zhao XD, Nampalli S, Serino AJ, Kumar S*. 2001. *Amersham Pharmacia Biotech Inc, 800 Centennial Ave, Piscataway, NJ 08855, USA. Immobilization of oligodeoxyribonucleotides with multiple anchors to microchips. *Nucleic Acids Res* **29:** (4) 955.

Zhumabayeva B, Chang C, McKinley J, Diatchenko L, Siebert PD. 2001. Clontech Labs Inc, 1020 East Meadow Circle, Palo Alto, Ca 94303, USA. Generation of full-length cDNA libraries enriched for differentially expressed genes for functional genomics. *Biotechniques* **30:** (3) 512.

### 16 Bioinformatics

Andre C, Vincens P, Boisvieux JF, Hazout S. 2001. Univ Paris 06, CHU Pitie Salpetriere, Dept Biomath, 91 Blvd Hop, FR-75634 Paris 13, France. MOSAIC: Segmenting multiple aligned DNA sequences. *Bioinformatics* **17:** (2) 196.

Baccam P, Thompson RJ, Fedrigo O, Carpenter S, Cornette JL. 2001. Los Alamos Nat Lab, MS K-710, Los Alamos, NM 87545, USA. PAQ: Partition analysis of quasispecies. *Bioinformatics* **17:** (1) 16.

Bejerano G, Yona G*. 2001. *Cornell Univ, Dept Comp Sci, Ithaca, NY 14853, USA. Variations of probabilistic suffix trees: Statistical modeling and prediction of protein families. *Bioinformatics* **17:** (1) 23.

Fariselli P, Casadio R*. 2001. *Univ Bologna, Dept Biol, CIRB Biocomputing Unit, Via Irnerio 42, IT-40126 Bologna, Italy. RCNPRED: Prediction of the residue co-ordination numbers in proteins. *Bioinformatics* **17:** (2) 202.

Frishman D, Albermann K, Hani J, Heumann K, Metanomski A, Zollner A, Mewes HW. 2001. Max-Planck-Inst Biochem, Munich Information Ctr Prot Sequences, DE-82152 Martinsried, Germany. Functional and structural genomics using PENDANT. *Bioinformatics* **17:** (1) 44.

Gemund C, Ramu C, Altenberg-Greulich B, Gibson TJ*. 2001. *European Mol Biol Lab, Postfach 102209, DE-69012 Heidelberg, Germany. Gene2EST: A BLAST2 server for searching expressed sequence tag (EST) databases with eukaryotic gene-sized queries. *Nucleic Acids Res* **29:** (6) 1272.

Hall D, Bhandarkar SM*, Wang J. 2001. *Univ Georgia, Dept Comp Sci, Boyd Grad Studies Res Ctr 415, Athens, Ga 30602, USA. ODS2: A multiplatform software application for creating integrated physical and genetic maps. *Genetics* **157:** (3) 1045.

Herrero J, Valencia A, Dopazo J*. 2001. *CNIO, Ctra Majadahonda-Pozuelo km 2, ES-28220 Madrid, Spain. A hierarchical unsupervised growing neural network for clustering gene expression patterns. *Bioinformatics* **17:** (2) 126.

Hu R, Wang B. 2001. Acad Sinica, Inst Theoret Phys, POB 2735, CN-10080 Beijing, Peoples Rep China. Statistically significant strings are related to regulatory elements in the promotor regions of *Saccharomyces cerevisiae*. *Physica A* **290:** (3-4) 464.

Huestis R, Cloonan N, Tchavtchitch M, Saul A. 2001. Monash Univ, Dept Microbiol, Clayton, Vic 3168, Australia. An algorithm to predict 3′ intron splice sites in *Plasmodium falciparum* genomic sequences. *Mol Biochem Parasitol* **112:** (1) 71.

Konno H, Fukunishi Y, Shibata K, Itoh M, Carninci P, Sugahara Y, Hayashizaki Y. 2001. RIKEN, Genomic Sci Ctr, Lab Genome Explorat Res Grp, Yokohama, Kanagawa 230 004, Japan. Computer-based methods for the mouse full-length cDNA encyclopedia: Real-time sequence clustering for construction of a nonredundant cDNA library. *Genome Res* **11:** (2) 281.

Letondal C. 2001. Inst Pasteur, Serv Informat Sci, 28 rue Dr Roux, FR-75724 Paris 15, France. A Web interface generator for molecular biology programs in Unix. *Bioinformatics* **17:** (1) 73.

Lexa M, Horak J, Brzobohaty B. 2001. Masaryk Univ, Fac Sci, Lab Plant Mol Physiol, Kotlarska 2, CZ-61137 Brno, Czech Republic. Virtual PCR. *Bioinformatics* **17:** (2) 192.

Li M, Badger JH, Chen X, Kwong S, Kearney P, Zhang HY. 2001. Univ Waterloo, Dept Comp Sci, Bioinformat Lab, Waterloo, Ontario, Canada N2L 3G1. An information-based sequence distance and its application to whole mitochondrial genome phylogeny. *Bioinformatics* **17:** (2) 149.

Martins RP, Leach RE, Krawetz SA*. 2001. *Wayne State Univ, Sch Med, Ctr Mol Med & Genet, 253 CS Mott Ctr, 275 East Hancock Ave, Detroit, Mi 48201, USA. Whole-body gene expression by data mining. *Genomics* **72:** (1) 34.

Micallef KP, Cooper M, Podlich DW. 2001. Univ Queensland, Sch Land & Food Sci, Brisbane, Qld 4072, Australia. Using clusters of computers for large QU-GENE simulation experiments. *Bioinformatics* **17:** (2) 194.

Mironov AA, Novichkov PS, Gelfand MS. 2001. State Sci Ctr Biotechnol NIIGenetika, RU-113545 Moscow, Russia. Pro-Frame: Similarity-based gene recognition in eukaryotic DNA sequences with errors. *Bioinformatics* **17:** (1) 13.

Mizuno H, Tanaka Y, Nakai K, Sarai A. 2001. Nippon Roche Res Ctr, 200 Kajiwara, Kanagawa 249 8530, Japan. ORI-GENE: gene classification based on the evolutionary tree. *Bioinformatics* **17:** (2) 167.

Ohya M, Sato K. 2000. Sci Univ Tokyo, Dept Information Sci, Noda, Chiba 278, Japan. Use of information theory to study genome sequences. *Rep Math Phys* **46:** (3) 419.

Ono T, Hishigaki H, Tanigami A, Takagi T*. 2001. *Univ Tokyo, Inst Med Sci, Ctr Human Genome, Minato ku, 4-6-1 Shirokanedai, Tokyo 108 8639, Japan. Automated extraction of information on protein-protein interactions from the biological literature. *Bioinformatics* **17:** (2) 155.

Patnaik SK, Blumenfeld OO. 2001. Albert Einstein Coll Med, Dept Cell Biol, Bronx, NY 10461, USA. Use of on-line tools and databases for routine sequence analyses. *Anal Biochem* **289:** (1) 1.

Raddatz G, Dehio M, Meyer TF, Dehio C. 2001. Max-Planck-Inst Biol, Infect Biol Abt, Spemannstr 34, DE-72076 Tubingen, Germany. PrimeArray: Genome-scale primer design for DNA-microarray construction. *Bioinformatics* **17:** (1) 98.

Ray WC, Daniels CJ. 2001. Ohio State Univ, Biophys Program, Columbus, Oh 43210, USA. The PACRAT system: An extensible WWW-based system for correlated sequence retrieval, storage and analysis. *Bioinformatics* **17:** (1) 100.

Roberts RJ, Varmus HE, Ashburner M, Brown PO, Eisen MB, Khosla C, Kirschner M, Nusse R, Scott M, Wold B. 2001. New England Biolabs Inc, 32 Tozer Rd, Beverly, Ma 01915, USA. Information access: Building a "GenBank" of the published literature. *Science* **291:** (5512) 2318.

Schaefer C, Grouse L, Buetow K, Strausberg RL*. 2001. *NCI, Canc Genomics Office Off, 31 Ctr Dr, Room 11A03, Bethesda, Md 20892, USA. A new cancer genome anatomy project web resource for the community. *Cancer J* **7:** (1) 52.

Selley JN, Swift J, Attwood TK. 2001. Univ Manchester, Sch Biol Sci, Oxford Rd, Manchester M13 9PT, England. EASY - An EXpert Analysis SYstem for interpreting database search outputs. *Bioinformatics* **17:** (1) 105.

Shapiro BA, Wu JC, Bengali D, Potts MJ. 2001. NIH/NCI, Image Proc Sect, Lab Expt & Computational Biol, Div Basic Sci, Frederick Canc Res & Dev Ctr, Frederick, Md 21702, USA. The massively parallel genetic algorithm for RNA folding: MIMD implementation and population variation. *Bioinformatics* **17:** (2) 137.

Siepel A, Farmer A, Tolopko A, Zhuang M, Mendes P, Beavis W, Sobral B. 2001. Natl Ctr Genome Resources, 2935 Rodeo Pk Dr East, Santa Fe, NM 87505, USA. ISYS: A decentralized, component-based approach to the integration of heterogeneous bioinformatics resources *Bioinformatics* **17:** (1) 83.

Spiers AJ, Field D, Bailey M, Rainey PB. 2001. Univ Oxford, Dept Plant Sci, Oxford, England. Notes on designing a partial genomic database: The PfSBW25 Encyclopaedia, a sequence database for *Pseudomonas fluorescens* SBW25. *Microbiology* **147:** (2) 247.

Copyright © 2001 John Wiley & Sons, Ltd.
